# Multiple AUX/IAA–ARF modules regulate lateral root formation: the role of *Arabidopsis* SHY2/IAA3-mediated auxin signalling

**DOI:** 10.1098/rstb.2011.0232

**Published:** 2012-06-05

**Authors:** Tatsuaki Goh, Hiroyuki Kasahara, Tetsuro Mimura, Yuji Kamiya, Hidehiro Fukaki

**Affiliations:** 1 Department of Biology, Graduate School of Science, Kobe University, 1-1 Rokkodai, Kobe 657-8501, Japan; 2 Plant Science Center, RIKEN, Yokohama, Kanagawa 230-0045, Japan

**Keywords:** auxin, lateral root formation, SHY2/IAA3, *Arabidopsis*

## Abstract

In *Arabidopsis thaliana*, lateral root (LR) formation is regulated by multiple auxin/indole-3-acetic acid (Aux/IAA)–AUXIN RESPONSE FACTOR (ARF) modules: (i) the IAA28–ARFs module regulates LR founder cell specification; (ii) the SOLITARY-ROOT (SLR)/IAA14–ARF7–ARF19 module regulates nuclear migration and asymmetric cell divisions of the LR founder cells for LR initiation; and (iii) the BODENLOS/IAA12–MONOPTEROS/ARF5 module also regulates LR initiation and organogenesis. The number of Aux/IAA–ARF modules involved in LR formation remains unknown. In this study, we isolated the *shy2-101* mutant, a gain-of-function allele of *short hypocotyl2/suppressor of hy2* (*shy2*)*/iaa3* in the Columbia accession. We demonstrated that the *shy2-101* mutation not only strongly inhibits LR primordium development and emergence but also significantly increases the number of LR initiation sites with the activation of *LATERAL ORGAN BOUNDARIES-DOMAIN16/ASYMMETRIC LEAVES2-LIKE18*, a target gene of the SLR/IAA14–ARF7–ARF19 module. Genetic analysis revealed that enhanced LR initiation in *shy2-101* depended on the SLR/IAA14–ARF7–ARF19 module. We also showed that the *shy2* roots contain higher levels of endogenous IAA. These observations indicate that the SHY2/IAA3–ARF-signalling module regulates not only LR primordium development and emergence after SLR/IAA14–ARF7–ARF19 module-dependent LR initiation but also inhibits LR initiation by affecting auxin homeostasis, suggesting that multiple Aux/IAA–ARF modules cooperatively regulate the developmental steps during LR formation.

## 1. Introduction

In vascular plants, branched root systems arise through the production of lateral and adventitious roots, thereby allowing the plants to absorb water and nutrients from the soil and to sustain the aerial shoots [1]. In most eudicot plants, lateral roots (LRs) initiate from asymmetric, anticlinal cell divisions in the xylem pole pericycle cells of the parental roots [1,2]. These divided pericycle cells undergo periclinal cell divisions to produce a young LR primordium. Subsequent cell divisions and cell differentiation lead to the establishment of a mature LR primordium with a root apical meristem. Finally, the LR emerges through the outer cortical and epidermal cell layers of parental roots [3,4]. Recent molecular genetic and physiological studies in several model plants have shown that LR formation is dependent on auxin [4–6]. In *Arabidopsis*, auxin regulates most of the developmental steps during LR formation; LR founder cell specification, LR initiation, LR primordium development and LR emergence [4–9].

In plant cells, auxin regulates transcription of many auxin-responsive genes dependent on auxin/indole-3-acetic acid (Aux/IAA)–AUXIN RESPONSE FACTOR (ARF) auxin-signalling modules [10]. In *Arabidopsis*, there are 22 functional ARFs and 29 Aux/IAA proteins [11,12]. ARFs directly activate or repress the transcription of their target genes that contain AuxREs, auxin responsive elements in the promoter. In the absence of auxin, the Aux/IAA protein interacts with its partner ARF, thereby inactivating ARF activity. In the presence of auxin, the Aux/IAA protein is degraded through ubiquitination by the SCF^TIR1/AFBs^ E3 ubiquitin ligase complex that contains the auxin receptor TIR1/AFBs [13–16]. Activated ARFs positively or negatively regulate target genes, resulting in ARF-dependent auxin responses. Gain-of-function mutations in domain II of Aux/IAAs stabilize the protein even in the presence of auxin, thereby constitutively inactivating ARF activity as well as affecting auxin-mediated growth and development [12,17]. ARFs and Aux/IAAs have distinct and overlapping functions in plant growth and development [18–23]. Particularly, molecular genetic studies with mutants defective in LR formation have shown that several Aux/IAA–ARF modules play important roles in the developmental steps during LR formation. For example (i) the IAA28–ARFs module regulates the LR founder cell specification in the root basal meristem [7]; (ii) the SOLITARY-ROOT (SLR)/IAA14–ARF7–ARF19 module regulates nuclear migration and asymmetric cell divisions of LR founder cells for LR initiation [9,18,24,25]; and (iii) the BODENLOS (BDL)/IAA12-MONOPTEROS (MP)/ARF5 module also participates in LR initiation after the SLR/IAA14–ARF7–ARF19 module acts [10,26]. Among these auxin-signalling modules, the SLR/IAA14–ARF7–ARF19 module regulates LR initiation by activating several auxin-responsive genes [9,18,20,24,25,27]. The gain-of-function *slr-1* mutation in the *SLR/IAA14* gene blocks pericycle cell division for LR initiation, resulting in a solitary-root phenotype [9,27]. In contrast, the *arf7 arf19* loss-of-function double mutant also has a few LRs, but the *arf7* and *arf19* single mutants do produce LRs, indicating that ARF7 and ARF19 have redundant functions for LR formation [18,20]. The *SLR/IAA14*, *ARF7* and *ARF19* genes are co-expressed in root tissues, including the pericycle, and SLR/IAA14 interacts with ARF7 and ARF19 in a yeast two-hybrid system [18,24]. These results strongly suggested that the stabilized mutant IAA14 constitutively inhibits the activity of ARF7 and 19, thereby repressing the downstream genes for LR initiation. Therefore, auxin was proposed to promote the degradation of SLR/IAA14 and the other Aux/IAAs, resulting in the activation of ARF7/19-dependent transcription of the target genes involved in LR initiation [18,20,24]. In fact, ARF7 and ARF19 were recently shown to regulate LR initiation via activating *LATERAL ORGAN BOUNDARIES-DOMAIN (LBD)/ASYMMETRIC LEAVES2-LIKE* (*ASL*) genes such as *LBD16/ASL18* [25].

At present, the number of Aux/IAA–ARF modules involved in LR formation is unknown. In addition to the mutations in IAA28, SLR/IAA14 and BDL/IAA12, gain-of-function mutations in other Aux/IAA members, including AUXIN RESISTANT5 (AXR5)/IAA1, SHORT HYPOCOTYL2/SUPPRESSOR OF HY2 (SHY2)/IAA3, CRANE/IAA18, MASSUGU2 (MSG2)/IAA19 also decrease the number of LRs, indicating that auxin signalling dependent on these Aux/IAAs is necessary for LR formation [6,28]. The *aux/iaa* mutants do have phenotypic differences in LR formation. For example, the *slr-1* mutant has no LRs [9], whereas the other mutants including *axr5/iaa1*, *shy2/iaa3*, *crane/iaa18*, *msg2/iaa19* and *iaa28* have a decreased number of LRs but retain the ability to form LRs [9,29–34]. In addition, because these *aux/iaa* mutants were isolated and characterized by several laboratories using different growth conditions, how their LR phenotypes differ in terms of the frequency of LR initiation, the positioning of LRs and the emergence of LRs is unknown. Furthermore, because a few of the *aux/iaa* mutants (*shy2-2*, *shy2-3* and *iaa28-1*) are isolated in genetic backgrounds other than the Columbia (Col) accession, it is necessary to carefully characterize and compare the LR phenotype among the *aux/iaa* mutants isolated from these different accessions.

Previous studies on the *shy2-2/iaa3* allele, isolated in the Landsberg *erecta* (L*er*) accession, have shown that SHY2/IAA3-mediated auxin signalling is important for LR emergence because *shy2-2* has a decreased number of emerged LRs and an increased number of non-emerged LR primordia. In contrast, *shy2-24*, a loss-of-function allele, has an increased number of emerged LRs and a decreased number of non-emerged LR primordia. These results indicate that SHY2/IAA3 negatively regulates LR emergence [30,35]. Expression analyses using the *SHY2* promoter-GUS line have shown that *SHY2/IAA3* is expressed in the root endodermis [35], indicating that SHY2/IAA3-mediated auxin signalling for LR emergence occurs in the endodermal tissue. However, how SHY2/IAA3-mediated auxin signalling affects LR initiation and interacts with the other Aux/IAA–ARF modules such as the SLR/IAA14–ARF7–ARF19 module during LR formation is unknown.

In this study, we isolated the *shy2-101* mutant, a new gain-of-function allele of *shy2/iaa3* in the Col accession background, and characterized the LR phenotype in detail. We demonstrated that the *shy2-101/iaa3* mutation strongly inhibited LR primordium development and LR emergence as observed in the *shy2-2* mutant in the L*er* accession background, but the *shy2-101/iaa3* mutation significantly increased LR initiation sites with the activation of *LBD16/ASL18*, a target gene of the SLR/IAA14–ARF7–ARF19 module. Genetic analysis revealed that the enhanced LR initiation in the *shy2-101* mutant depended on the SLR/IAA14–ARF7–ARF19 module. In addition, we showed that the *shy2* mutations strongly affect auxin homeostasis in the roots. Our results indicate the critical role of the SHY2/IAA3–ARFs module in LR formation after SLR/IAA14–ARF7–ARF19-dependent LR initiation, suggesting that multiple Aux/IAA–ARF-signalling modules cooperatively regulate the developmental steps during LR formation.

## 2. Material and methods

### (a) Plant materials and growth conditions

*Arabidopsis thaliana* accessions Columbia (Col-0) and Landsberg *erecta* (L*er*) were used in this study. The *shy2-101* mutant line was isolated as a mutant with fewer LRs from ethyl methanesulphonate (EMS)-mutagenized M_2_ Col seeds that were purchased from LEHLE SEEDS (http://www.arabidopsis.com/). The *slr-1*, *arf7-1*, *arf19-1* and *pLBD16::GUS* lines have been described previously [18,25]. The *shy2-2* mutant seeds (L*er* accession) were kindly provided by Jason W. Reed (University of North Carolina, USA) [30]. Seeds were germinated under sterile conditions on 1× Murashige–Skoog medium with 1 per cent sucrose. Plants were grown at 23°C under continuous light as described previously [9]. The number of LRs and root length were determined using a dissecting microscope and ImageJ software (NIH).

### (b) Microscopy

β-Glucuronidase (GUS) staining, fixation and whole-mount clearing of roots were performed essentially as described earlier [3], and samples were observed with a Leica DM6000 microscope equipped with Nomarski optics (Leica Microsystems, Wetzlar, Germany).

### (c) LC–ESI–MS/MS analysis of indole-3-acetic acid

IAA analysis was performed as described by Mashiguchi *et al.* [36] with slight modifications. For analysis of IAA in *Arabidopsis* seedlings, fresh plant tissues (30–40 mg) were homogenized in 80 per cent acetone/H_2_O (0.5–1 ml) containing ^13^C_6_-IAA (Cambridge Isotope Laboratories), and purified by high performance liquid chromatography as described previously [37]. The IAA fraction was redissolved in 10 per cent methanol/H_2_O with 1 per cent acetic acid (1 ml) and fractionated to an Oasis HLB column (1 cc; Waters). The column was washed with 20 per cent methanol/H_2_O with 1 per cent acetic acid (1 ml), and IAA was eluted with 70 per cent methanol/H_2_O with 1 per cent acetic acid (1 ml) and evaporated to dryness by using a Speed-Vac. The IAA fraction was redissolved in 1 per cent acetic acid/H_2_O (10–20 μl) and injected into an LC–ESI–MS/MS. MS/MS analysis conditions were as follows: capillary, 3.00 kV; source temperature, 100°C; desolvation temperature, 500°C; collision energy, 10 V; sampling cone voltage, 16 V; scan time, 0.6 s per scan (delay, 0.05 s); and MS/MS transition (*m*/*z*), 176.1/130.1 for unlabelled IAA and 182.1/136.1 for ^13^C_6_-IAA, respectively. Ultra-performance liquid chromatography conditions and quantification of IAA were the same as in previous methods [37].

## 3. Results

### (a) The *shy2-101* mutation inhibits lateral root primordium development and lateral root emergence but increases the number of lateral root founder cells

**Figure 1.**
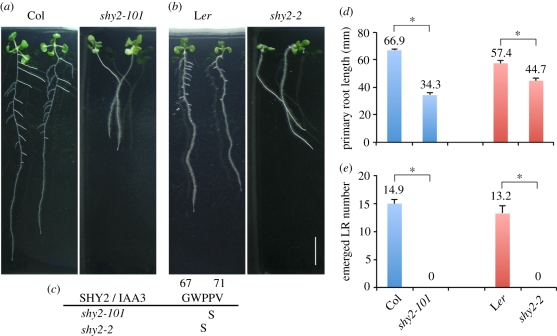
Lateral root (LR) formation is inhibited by the gain-of-function *shy2/iaa3* mutants. (*a*,*b*) 10-day-old seedlings of Col and *shy2-101* (*a*), and those of L*er* and *shy2-2* (*b*). Scale bar = 10 mm. (*c*) Amino acid sequences of the conserved region in domain II of the SHY2/IAA3 protein. The *shy2-101* mutation changes the 70th amino acid from proline to serine (P70S), whereas the *shy2-2* mutation changes the 69th amino acid from proline to serine (P69S) [30]. (*d*,*e*) Primary root length (*d*) and emerged LR number (*e*) of Col, *shy2-101*, L*er* and *shy2-2* seedlings at 10 days after germination (*n* = 15). Error bars indicate standard error of the mean. Asterisks indicate a statistical difference (*p < 0.01 by a two-sided *t*-test).

The *shy2-101* mutant was screened from EMS-mutagenized Col M_2_ seedlings and selected based on having fewer LRs than wild-type (figure 1*a*). Genetic and sequence analyses showed that *shy2-101* has a gain-of-function mutation in domain II of IAA3 that changes the 70th amino acid from proline to serine (P70S), thereby stabilizing the IAA3 protein in a manner similar to the *shy2-2* mutation [38] (figure 1*c*). As observed in the other gain-of-function *shy2* mutants (*shy2-2*, *shy2-3*) isolated from the L*er* background [30], *shy2-101* showed a dwarf shoot phenotype with curled-up leaves and an abnormal gravitropic response in the root (data not shown). Because most of the auxin-related mutants and reporter lines in *Arabidopsis* are produced in the Col accession, the *shy2-101* allele will be useful in comparing phenotype and reporter expression with other mutants isolated in the Col background as described below.

First, we compared the primary root length and number of LRs in 10-day-old wild-type Col and *shy2-101* seedlings grown under standard conditions (constant light, 1× MS medium). Compared with wild-type Col, the primary root length of *shy2-101* seedlings was shorter (figure 1*a*,*d*)*.* In addition, *shy2-101* seedlings had no emerged LRs, whereas the wild-type seedlings had many LRs (figure 1*a*,*e*). These *shy2-101* phenotypes were similar to those of the *shy2-2* mutant in L*er* (figure 1*b*,*d*,*e*); however, the degree of primary root growth inhibition was higher in *shy2-101* (root growth was 51.3% of Col root length) than in the *shy2-2* (root growth was 77.9% of L*er* root length), suggesting an accession-specific effect on the *shy2* mutant phenotype. The LR density (number of emerged LR per portion of the primary root where LRs are present) was zero in both *shy2* mutant alleles, indicating that SHY2/IAA3-mediated auxin signalling is important for LR formation.

**Figure 2.**
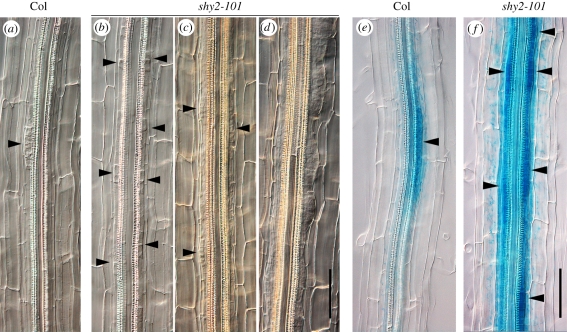
The *shy2-101* mutation inhibits lateral root (LR) primordium development but increases the number of LR founder cells expressing *pLBD16::GUS* reporter. (*a*–*d*) Nomarski images of Col (*a*) and *shy2-101* (*b*–*d*) primary roots at 12 days after germination (DAG). Three panels of *shy2-101* (*b*–*d*) show the different mature root regions, respectively: distal (*b*), proximal (*c*) and more proximal regions (*d*). Black arrowheads indicate LR primordia. Scale bar = 100 µm. (*e*,*f*) Expression of *pLBD16::GUS* in the mature root region of Col (*e*) and *shy2-101* (*f*) seedlings at 7 DAG. Black arrowheads indicate LR initiation sites expressing *pLBD16::GUS*. Scale bar = 100 µm.

Next, we examined whether LR primordium formation occurred in the *shy2-101* mutant. The 12-day-old seedlings were cleared, and non-emerged LR primordia were observed microscopically. In the Col, a few LR initiation sites were observed in the distal primary root region where asymmetric cell divisions occur (figure 2*a*). In contrast, the *shy2-101* roots had more LR initiation sites in the same distal root region but subsequent periclinal cell divisions were inhibited (figure 2*b*). Interestingly, in the proximal regions of the *shy2-101* primary root, many early-stage LR primordia were observed but LR emergence was significantly inhibited (figure 2*c*,*d*). These results strongly suggest that the *shy2-101* mutation increases the number of LR initiation events with anticlinal cell divisions of xylem pole pericycle, but inhibits/retards subsequent LR primordium development and LR emergence. Similar LR phenotypes were also observed in *shy2-2* (data not shown), confirming that SHY2/IAA3-mediated auxin signalling is important for LR primordium development and LR emergence in both Col and L*er* genetic backgrounds.

### (b) The *shy2-101* mutation increases the number of lateral root initiation sites expressing *LBD16/ASL18*, a key gene for lateral root initiation

To determine whether the *shy2-101* mutation increases the activity of LR initiation genes, we monitored the expression of the *pLBD16::GUS* reporter that was constructed in the Col background [25]. *LBD16/ASL18* is one of the direct target genes activated by ARF7 and ARF19 and functions in LR formation downstream of ARF7/ARF19 [25]. In 7-day-old wild-type Col seedlings, *pLBD16::GUS* was specifically expressed in the LR founder cells and LR initiation sites along the xylem pole pericycle (figure 2*e*) [25]. This strong GUS activity was never observed in the *slr-1/iaa14* mutant (Goh *et al.* 2011, unpublished results). Surprisingly, 7-day-old *shy2-101* seedlings already had many LR initiation sites with *pLBD16::GUS* activity (figure 2*f*). These results indicate that the *shy2-101* mutation significantly increases LR initiation events with the activation of *LBD16*, suggesting that SHY2/IAA3-mediated auxin signalling is necessary for inhibiting LR initiation in the pericycle cells.

**Figure 3.**
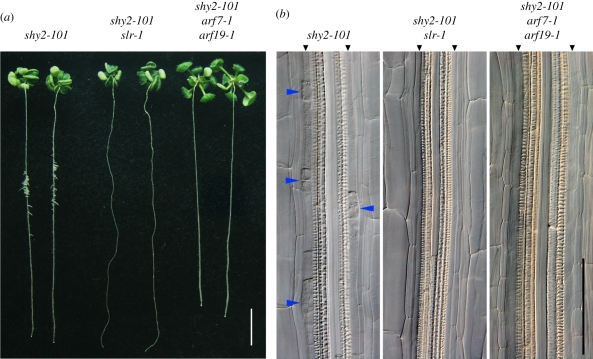
Genetic interactions between *shy2-101* and *slr-1*, *arf7-1* and *arf19-1*. (*a*) 15-day-old seedlings of *shy2-101*, *shy2-101 slr-1*, and *shy2-101 arf7-1 arf19-1* mutants. Scale bar = 10 mm. (*b*) Nomarski images of roots from *shy2-101*, *shy2-101 slr-1*, and *shy2-101 arf7-1 arf19-1* mutants at 9 days after germination. Enhanced lateral root (LR) initiation in *shy2-101* is blocked by the *slr-1* mutation or the *arf7 arf19* double mutation. Blue arrowheads indicate LR initiation sites, and black arrowheads show pericycle cell layers adjacent to the xylem pole. Scale bar = 100 µm.

The *shy2-101* mutation also inhibited LR primordium development and LR emergence after LR initiation in 12-day-old seedlings (figure 2), whereas at 15 days post-germination the *shy2-101* mutation often caused ‘clustered LRs’, where numerous LRs emerged from a limited region of the primary root (figure 3*a*). We hypothesize that these clustered LRs are already initiated at a younger age and eventually develop LR primordia and emerge at an older age.

### (c) Enhanced lateral root initiation in *shy2-101* is dependent on activation of the SLR/IAA14–ARF7–ARF19 auxin-signalling module

The SLR/IAA14–ARF7–ARF19 auxin-signalling module is necessary for LR initiation [9,18,20,24,25]. To determine whether enhanced LR initiation in *shy2-101* depends on the SLR/IAA14–ARF7–ARF19 auxin-signalling module, we constructed the *shy2-101 slr-1* double and *shy2-101 arf7-1 arf19-1* triple mutants in the Col background, and analysed their LR phenotype. As shown in figure 3, the *shy2-101 slr-1* double-mutant seedlings had no LR initiation sites, resulting in the *slr-1*-like LR phenotype in 15-day-old seedlings (figure 3*a*,*b*). These observations indicate that the *slr-1* mutation blocks the enhanced LR initiation in *shy2-101*. Similarly, the 15-day-old *shy2-101 arf7-1 arf19-1* triple-mutant seedlings had no LRs, whereas the *shy2-101* seedlings had clustered LRs (figure 3*a*). There were no LR initiation sites in either *shy2-101 slr-1* or *shy2-101 arf7-1 arf19-1* primary roots (figure 3*b*). These results also indicate that the enhanced LR initiation in the *shy2-101* mutant is dependent on activation of the SLR/IAA14–ARF7–ARF19 signalling module, suggesting that the *shy2-101* mutation acts on LR formation downstream of SLR/IAA14–ARF7–ARF19 module-dependent LR initiation.

### (d) The *shy2* mutations strongly elevate endogenous indole-3-acetic acid level in the roots

**Figure 4.**
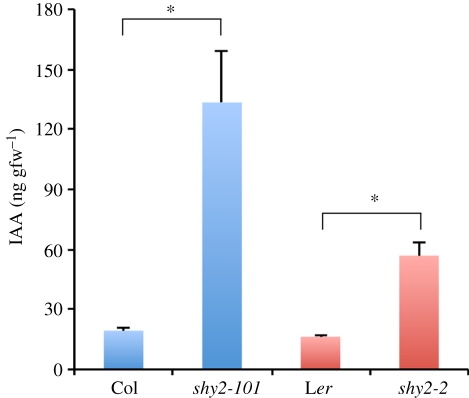
Indole-3-acetic acid (IAA) levels in wild-type and *shy2* mutant plants. IAA levels (ng gfw^−1^) in the roots of the 7-day-old wild-type (Col and L*er*) and *shy2* (*shy2-101* and *shy2-2*) mutant seedlings are shown (*n* = 4). Error bars indicate standard error of the mean. Asterisks indicate a statistical difference (*p < 0.01 by a two-sided *t*-test). Experiments were repeated twice, and similar values were obtained in each experiment.

Our genetic evidence that enhanced LR initiation in the *shy2-101* mutant depends on the SLR/IAA14–ARF7–ARF19 auxin-signalling module suggests the possibility that the *shy2* mutant might contain higher auxin levels in the roots, thereby increasing the number of LR initiation sites. To clarify this point, we measured endogenous IAA levels in the roots of 7-day-old wild-type and *shy2* seedlings by LC–ESI–MS/MS analysis (see §2). As shown in figure 4, both the *shy2-101* (in Col background) and *shy2-2* (in L*er* background) mutants contained a higher level of endogenous IAA in the roots, compared with their corresponding wild-types. Particularly, the *shy2-101* roots had a much higher IAA level than *shy2-2*, suggesting differences in the genetic background between the L*er* and Col accessions (figure 4). These results indicate that the *shy2* gain-of-function mutations increased endogenous auxin levels in roots, strongly suggesting that elevated auxin levels promoted LR initiation and resulted in the increased number of LR initiation sites in the *shy2-101* mutant.

## 4. Discussion

**Figure 5.**
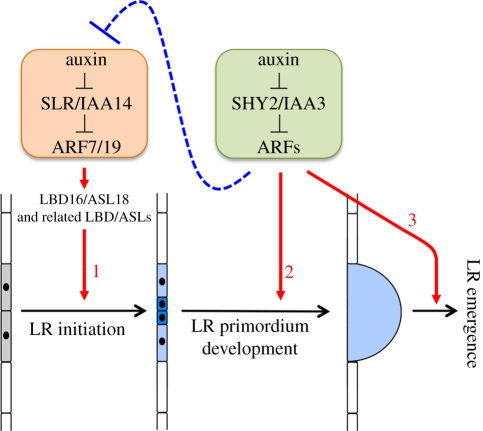
Schematic of lateral root (LR) formation regulated by SLR/IAA14–ARF7–ARF19 and SHY2/IAA3–ARFs auxin-signalling modules. LR initiation is controlled by the SLR/IAA14–ARF7–ARF19 auxin-signalling module (orange box) by the activation of LBD16/ASL18 and its related LBD/ASL proteins (red arrow 1). After initiation, the SHY2/IAA3–ARFs signalling module (green box) plays a role not only for LR primordium development and LR emergence after the SLR/IAA14–ARF7–ARF19 module (red arrows 2 and 3) but also for inhibition of SLR/IAA14–ARF7–ARF19-dependent LR initiation in the xylem pericycle cells by affecting auxin homeostasis (blue dotted line).

In this study, we demonstrated that the *shy2-101* mutation, a newly isolated gain-of-function mutant of *shy2/iaa3* in the Col genetic background, strongly inhibits LR primordium development and LR emergence as well as also significantly increases LR initiation events with the activation of *LBD16/ASL18*, a key gene for LR initiation. Previous studies have reported that the *shy2-2* mutant, isolated in the L*er* background, also has a decreased number of emerged LRs and an increased number of non-emerged LR primordia, whereas *shy2-24*, a loss-of-function allele, has an increased number of emerged LRs and a decreased number of non-emerged LR primordia [35]. We observed that the *shy2-101* mutation in the Col background also inhibits LR primordium development and LR emergence, showing a LR phenotype similar to that of *shy2-2* in spite of their being in different genetic backgrounds. These results indicate that the SHY2/IAA3–ARFs module regulates LR primordium development and LR emergence (figure 5). Previous expression analyses have shown that *SHY2/IAA3* is expressed in the root endodermis, where *LAX3*, an auxin influx carrier required for LR emergence, is also co-expressed [35]. A model was proposed in which auxin originating from the dividing xylem pole pericycle cells induces cell wall-remodelling gene expression in the adjacent endodermal cells by targeting the degradation of SHY2/IAA3, allowing the LR primordium to emerge through the outer cortex and epidermis by inducing LAX3 in the cortex and epidermis [35]. As the *shy2-2* mutation is also thought to inhibit the SHY2/IAA3-mediated induction of cell wall-remodelling genes in the endodermis [35], it is possible that cell wall remodelling in the adjacent endodermal cells may produce a signal for the early LR primordium to stimulate subsequent LR primordium development and LR emergence. The *shy2* mutation inhibits the expression of many kinds of genes [39], suggesting that the SHY2/IAA3–ARFs module positively regulates the genes necessary for LR primordium development and LR emergence (figure 5).

In addition to the inhibition of LR primordium development and emergence, the *shy2-101* mutation significantly increased the number of LR initiation sites expressing *pLBD16::GUS* (figure 2), suggesting that the *shy2-101* mutation enhanced the auxin response in the xylem pole pericycle, thereby increasing the number of LR founder cells. As hypothesized by Swarup *et al.* [35], shoot-derived auxin may hyper-accumulate in the root pericycle of the *shy2-101* mutant, resulting in more LR initiation sites compared with the wild-type. In fact, we demonstrated that the *shy2-101* and *shy2-2* mutants contained higher levels of endogenous IAA in the roots (figure 4), strongly suggesting that the elevated auxin promoted LR initiation and resulted in the increased number of LR initiation sites in the *shy2-101* mutant. The exact mechanism for SHY2/IAA3-mediated auxin homeostasis is unknown, but the *shy2-2* mutation reduces the expression of several *GH3* genes (*GH3-3* and *GH3-5*) that are involved in the control of auxin homeostasis [39,40], suggesting that normal SHY2/IAA3-mediated auxin signalling negatively controls the number of LR initiation sites by decreasing the free IAA level in the roots. Alternatively, the possibility still remains that a newly initiated young LR primordium, or LR primordium development itself may act as a repressor of LR initiation in the adjacent pericycle cells, but the *shy2-101* mutation may block such lateral inhibition of LRs. Transgenic plants expressing the stabilized bdl/iaa12 protein under the regulation of the *BDL/IAA12* promoter, which is active in dividing pericycle cells, had clustered roots as a result of ectopic pericycle cell divisions during LR initiation as observed in the weak *mp/arf5* mutant allele [10]. However, the *shy2-101* phenotype is different from that of the bdl/iaa12-expressing plants because the *shy2-101* mutation did not cause an aberrant pericycle cell division pattern during LR initiation (figure 2). Considering that *SHY2/IAA3* is expressed in the endodermis [35], our results suggest that the SHY2/IAA3–ARFs module regulates the inhibition of SLR/IAA14–ARF7–ARF19-dependent LR initiation indirectly by affecting auxin homeostasis in the roots (figure 5).

We also observed phenotypic differences in both primary root growth and endogenous IAA levels between the *shy2-2* (in L*er* background) and *shy2-101* (in Col background) mutants that may be due to differences in the genetic background between the L*er* and Col accessions. We hypothesize that the enhanced *shy2* inhibitory effect on primary root growth in the Col background might be due to the increased IAA level in the *shy2-101* (in Col background) roots. Such accession-dependent effects on mutant phenotypes are also observed in the *crane/iaa18* mutants; the *iaa18-1* allele in the L*er* background had defects in embryonic patterning, whereas the *crane-2/iaa18* allele in the Col background had almost no effect on embryonic patterning [33,34]. Recent studies using the *shy2-2* mutant have shown that primary root growth of *Arabidopsis* is regulated by complicated cross-talk among plant hormones, including auxin, cytokinin and gibberellin, whereas SHY2/IAA3 plays a key role for cell differentiation and division balance necessary for controlling root meristem size and root growth [41,42]. Thus, the *shy2-101* allele in the Col background may be helpful for studying accession-dependent effects on root growth regulation.

SHY2/IAA3 has been hypothesized to pair with ARF7 and ARF19 for the auxin response in the root gravitropic response, because both the *arf7 arf19* and *shy2* mutants are defective in the root gravitropic response [43]. In addition, SHY2/IAA3 interacts with ARF19 in a yeast two-hybrid system [43]. However, because the LR phenotype of the *shy2* mutants is different from that of the *arf7 arf19* mutant in which LR initiation is inhibited [18,25], it is unclear whether SHY2/IAA3 forms pairs with ARF7 and ARF19 during LR formation. Because *SHY2/IAA3* is expressed in the root endodermis [35], the corresponding ARFs should also be expressed in the endodermis. *ARF19* is ubiquitously expressed in the root [18], suggesting the possibility that the SHY2/IAA3–ARF19 module might regulate LR formation.

## 5. Conclusions and outlook

Recent molecular genetic studies of the gain-of-function mutants in the Aux/IAA family and loss-of-function mutants in ARFs have revealed that the developmental events during LR formation are regulated by multiple Aux/IAA–ARF auxin-signalling modules. Before LR initiation, the LR founder cell specification occurs in the root basal meristem, which is regulated by the IAA28–ARFs module [7]. Then, nuclear migration and asymmetric cell divisions of the LR founder cells lead to LR initiation that depends on the SLR/IAA14–ARF7–ARF19 module [18,24,25,44]. In addition, the BDL/IAA12-MP/ARF5 module also regulates LR initiation and LR organogenesis by repressing ectopic pericycle cell divisions [10]. In addition to these three modules, the *shy2-101/iaa3* LR phenotype strongly suggests that a fourth auxin-signalling module, SHY2/IAA3–ARFs, plays a role not only for LR primordium development and LR emergence but also for inhibition of LR initiation through affecting auxin homeostasis (figure 5). Identification of the corresponding ARFs for SHY2/IAA3-mediated signalling in LR formation and further study of the downstream genes will reveal the SHY2/IAA3–ARFs module-dependent molecular cascade that regulates LR initiation, LR primordium development and LR emergence. Furthermore, it will be important to determine the roles of the other Aux/IAA–ARF modules in LR formation, thereby contributing to our understanding of the mechanism that regulates plant root formation by multiple Aux/IAA–ARF auxin-signalling modules.
